# Combatting joint pain and inflammation by dual inhibition of monoacylglycerol lipase and cyclooxygenase-2 in a rat model of osteoarthritis

**DOI:** 10.1186/s13075-020-2096-3

**Published:** 2020-01-14

**Authors:** Holly T. Philpott, Jason J. McDougall

**Affiliations:** Departments of Pharmacology and Anaesthesia, Pain Management & Perioperative Medicine, Dalhousie University, 5850 College Street, Halifax, Nova Scotia B3H 4R2 Canada

**Keywords:** Cannabinoids, Osteoarthritis, Pain, Inflammation, Neuropathy, Coxib

## Abstract

**Background:**

Endocannabinoids are showing great promise as effective mediators for controlling joint inflammation and pain. One strategy that could be harnessed to promote endogenous cannabinoid function is to inhibit the enzymatic break down of endocannabinoids locally in the joint. KML29 is an inhibitor of monoacylglycerol lipase (MAGL) activity which has been shown to promote increased 2-arachodonylglycerol (2-AG) levels in the circulation and in peripheral tissues. It is also known that 2-AG can be metabolised via the cyclo-oxygenase-2 (COX-2) pathway leading to the production of pro-inflammatory prostaglandins, which may counteract the effects of 2-AG. Therefore, this study examined the effect of KML29 alone as well as in combination with low-dose celecoxib (CXB) on joint pain and inflammation in the monoiodoacetate (MIA) model of osteoarthritis (OA) pain.

**Methods:**

Injection of MIA (3 mg) into the knee joints of male Wistar rats was used to model OA pain, inflammation, and nerve damage. Pain behaviour was assessed by von Frey hair algesiometry, and inflammation was evaluated using intravital microscopy to measure leukocyte trafficking in the synovial microvasculature.

**Results:**

Intra-articular injection of MIA produced mechanical hypersensitivity as measured by von Frey hair algesiometry. Local injection of KML29 (700 μg) reduced joint pain at day 14 post-MIA induction, and this analgesic effect was blocked by the cannabinoid receptor antagonists AM281 and AM630 (*P* < 0.0001; *n* = 6). During the acute inflammatory phase of the MIA model (day 1), a significant reduction in withdrawal threshold (*P* < 0.0001; *n* = 6–8) and leukocyte trafficking was seen after treatment with KML29 + CXB (*P* < 0.0001; *n* = 6–8). Early treatment of MIA-injected knees (days 1–3) with KML29 + CXB ameliorated the development of mechanical secondary allodynia (*P* < 0.0001; *n* = 8) in the later stages of the MIA model.

**Conclusions:**

Combination therapy of KML29 plus CXB reduced joint pain and inflammation. Thus, dual inhibition of MAGL and cyclooxygenase-2 pathways could be a useful approach to alleviate joint inflammation and pain in OA joints.

## Background

Osteoarthritis (OA) is a disease of synovial joints characterised by tissue degeneration, pain, and intermittent low-grade inflammation [1]. During inflammatory flares, leukocytes accumulate in the joint where they release various chemical mediators that contribute to joint damage and pain [2]. Although many patients are treated with first-line therapies such as non-steroidal anti-inflammatory drugs (NSAIDs), their long-term use is often hindered by adverse side effects, such as hepato- and cardio-toxicity, as well as gastrointestinal ulceration [3, 4].

One way to successfully alleviate pain and inflammation related to OA is by harnessing the endocannabinoid system (ECS). The ECS consists of the two main ligands, anandamide (AEA) and 2-arachidonoylglycerol (2-AG), and two receptors, cannabinoid receptor 1 (CBR1) and cannabinoid receptor 2 (CBR2). Savinainen et al. (2001) demonstrated that 2-AG was a full agonist, with similar potency, at both CBR1 and CBR2 and that AEA is a potent agonist solely at the CBR1 [5]. Tissue damage causes endocannabinoid levels to rise; however, they are then rapidly degraded by the catabolic enzymes fatty acid amide hydrolase (FAAH) and monoacylglycerol lipase (MAGL), thereby limiting their therapeutic potential. Inhibitors of FAAH and MAGL may help promote endocannabinoid levels which would extend their bioactive effects. Modulating the ECS using MAGL inhibitors has shown promise pre-clinically. Systemic administration of a MAGL inhibitor improved hindlimb weight-bearing deficits and referred pain in the sodium monoiodoacetate (MIA) model of OA [6]. Additionally, inhibition of MAGL reduced paw oedema in the formalin [7] and carrageenan models of acute inflammation [8]. In pain studies, MAGL inhibition was found to reduce tactile allodynia related to both inflammatory pain as well as neuropathic pain [8]. Although the effects of systemically administered MAGL inhibitors have been previously assessed in a model of OA [6], their effect locally in the joint when targeting the ECS peripherally has not yet been investigated. Therefore, the first aim of this study was to assess the effectiveness of MAGL inhibition when administered locally into an OA joint.

In addition to 2-AG being broken down by MAGL in vivo, this endocannabinoid can also be metabolised via the cyclo-oxygenase-2 (COX-2) pathway leading to the formation of pro-inflammatory and pain-producing prostaglandins [9]. This suggests that overproduction of 2-AG levels as a result of MAGL blockade could also activate the COX-2 pathway to produce mediators that would counteract the analgesic effects of 2-AG in the joint. Thus, the second aim of this study was to assess if greater alleviation of joint pain and inflammation could be achieved by co-administering the MAGL inhibitor KML29 with a low dose of the selective COX-2 inhibitor celecoxib (CXB). Experiments were designed to block inflammation during the acute phase of the MIA model (days 1–3) to determine if this could alter pain at end-stage disease (day 14).

## Methods

### Animals

A total of 90 male Wistar rats (150–175 g upon arrival; Charles River, Quebec, Canada) were housed in ventilated racks at 22 ± 2 °C on a 12:12-h light:dark cycle (light-on from 7:00 to 19:00). All rats were permitted at least 1 week to acclimate following arrival at the animal care facility. Animals were housed in pairs, cages were lined with woodchip bedding, and animals were provided with standard lab chow and water *ad libitum*, as well as environmental enrichment. All experimental protocols were approved by the Dalhousie University Committee on the Use of Laboratory Animals, which acts in accordance with Animal Research: Reporting of In Vivo Experiments (ARRIVE) and the standards put forth by the Canadian Council for Animal Care.

### Sodium monoiodoacetate model of OA

Animals were deeply anaesthetised (2–4% isoflurane; 100% oxygen at 1 l/min), and depth of anaesthesia was confirmed by a lack of a response to a noxious toe pinch. The right knee joint was shaved, swabbed with 100% ethanol, and 50 μl of MIA (3 mg in saline) was injected into the joint space (intra-articular (i.artic.)). The knee was then manually extended and flexed for 30 s to disperse the solution throughout the joint.

### Assessment of pain behaviour

#### von Frey hair algesiometry

von Frey hair algesiometry was used as a measure of secondary allodynia. Alert, unanaesthetised animals were placed in a Plexiglass chamber with a metal mesh flooring which allowed access to the plantar surface of each hindpaw. After allowing the animals to acclimate until exploratory behaviour ceased (approximately 10 min), ipsilateral hindpaw mechanosensitivity was assessed using a modification of the Dixon up-down method [10]. A von Frey hair was applied perpendicular to the plantar surface of the ipsilateral hindpaw (avoiding the toe pads) until the hair was flexed; the filament was then held in place for 3 s. If there was a positive response (i.e. withdrawal, shaking, or licking of the hindpaw), the next lower strength hair was applied; if there was a lack of response, the next higher strength hair was applied up to a cut-off of 15 g bending force. The withdrawal threshold was determined using the following formula: 10^[*Xf* + *kδ*]^/10,000; where *Xf* is the value (in log units) of the final von Frey hair used, *k* is the tabular value for the pattern of the last six positive/negative responses, and *δ* is the mean difference (in log units) between the stimuli.

### Assessment of inflammation

Animals were deeply anaesthetised by an intraperitoneal (i.p.) injection of urethane (25% solution; 2 g/kg) and underwent surgical preparation as previously described [11].

#### Intravital microscopy

Intravital microscopy (IVM) was used to assess leukocyte-endothelial interactions within the microcirculation of the knee joint, as described previously [11, 12]. Two measures of leukocyte-endothelial interactions were used to assess articular inflammation: (i) the number of rolling leukocytes to pass an arbitrary line perpendicular to the venule in 1 min was counted and (ii) the number of adherent leukocytes within a 100-μm portion of the venule. Rolling leukocytes were defined as positively stained cells travelling slower than the surrounding blood flow, and adherent leukocytes were defined as positively stained cells that remained stationary for a minimum of 30 s.

### Experimental timelines

#### Acute treatment with a MAGL inhibitor

For acute pain studies, the animals underwent baseline von Frey hair mechanosensitivity testing as described above. Separate cohorts were treated on day 14 post-MIA with an i.artic. injection of either vehicle (50 μl) or the MAGL inhibitor KML29 (700 μg/50 μl). von Frey hair algesiometry measurements for these experiments were conducted at 30, 60, 120, 180, and 240 min following drug administration. In separate groups, day 14 MIA rats were treated first with either the CBR1 antagonist, AM281 (75 μg/50 μl), the CBR2 antagonist, AM630 (75 μg/50 μl), or vehicle (50 μl) applied locally (subcutaneously (s.c.)) over the joint 10 min prior to i.artic. injection of KML29 (700 μg/50 μl). Secondary allodynia assessments were performed at 30, 60, 120, 180, and 240 min following KML29 administration.

#### Acute treatment with a selective COX-2 inhibitor

To assess the effects of COX-2 inhibition on OA-associated pain, a separate cohort of animals underwent von Frey hair mechanosensitivity testing on day 1 post-MIA injection, which corresponds to the peak of OA-associated inflammation in this model. This cohort of animals was split into three treatment groups to create a dose response for the selective COX-2 inhibitor, CXB (3 mg/kg, 10 mg/kg, or 30 mg/kg). Behavioural pain testing was performed at 30, 60, 120, 180, and 240 min post-drug administration.

Intravital microscopy was also carried out on day 1 post-MIA induction. For all treatment cohorts, recordings were taken at 360 min post-drug administration after the animals had previously completed behavioural testing.

#### Acute treatment with a combination of MAGL and COX-2 inhibitors

To investigate the effects of combining an endocannabinoid enhancing compound (KML29) with a sub-clinical dose of CXB, animals underwent baseline von Frey hair algesiometry measurements. One day post-MIA induction, the animals were again separated into three treatment groups: KML29 (700 μg/50 μl), CXB (3 mg/kg), or combination (KML29 + CXB). Pain assessments were conducted at 30, 60, 120, 180, and 240 min post-drug administration.

Inflammation measures were conducted for all experimental cohorts, and IVM recordings were taken at 360 min post-drug administration after the animals had previously completed the behavioural testing.

#### Prophylactic treatment with MAGL and COX-2 inhibitors

To investigate the effects of early treatments on end-stage OA pain, a group of rats were treated with either KML29 (700 μg/50 μl), CXB (3 mg/kg), a combination (KML29 + CXB), or vehicle (DMSO:cremaphor:saline). A single administration was given on days 1, 2, and 3 after the induction of MIA. Behavioural pain measurements were conducted on days 0, 1, 2, 3, 7, 10, and 14.

### Drugs and reagents

KML29 (MAGL inhibitor; 1-piperidinecarboxylic acid, 4-[bis(1,3-benzodioxol-5-yl)hydroxymethyl]-, 2,2,2-trifluoro-1-(trifluoromethyl)ethyl ester) was obtained from Med Chem Express Ltd. (Monmouth Junction, NJ, USA). Celecoxib (CXB; COX-2 inhibitor; 4-[5-(4-methylphenyl0-3-(trifluoromthyl)-1H-pyrazol-1-yl]-benzenesulfonamide), AM281 (CB1 receptor antagonist; 1-(2,4-dichlorophenyl)-5-(4-iodophenyl)-4-methyl-*N*-4-morpholinyl-1H-pyrazole-3-carboxamide), and AM630 (CB2 receptor antagonist; 6-iodo-2-methyl-1-(2-morpholin-4-ylethyl)indol-3-yl]-(4-methoxyphenyl)methanone) were obtained from Cayman Chemicals (Ann Arbor, MI, USA). Rhodamine 6G, cremophor, dimethyl sulphoxide (DMSO), urethane, and sodium monoiodoacetate (MIA) were obtained from Sigma Aldrich (St. Louis, MO, USA). Solutions of KML29, AM281, AM630, and celecoxib were prepared in vehicle (1:1:18; DMSO:cremophor:saline) on the day of use. Rhodamine 6G (0.05%) and MIA were dissolved in saline. Physiological buffer (135 mM NaCl, 20 mM NaHCO_3_, 5 mM KCl, 1 mM MgSO_4_·7H_2_O, pH = 7.4) was prepared in the lab.

### Statistical analysis

All data were expressed as mean ± standard error of the mean (SEM). Data were tested for Gaussian distribution by the Kolmogorov-Smirnov test. All data were normally distributed and were therefore analysed using parametric statistics (two-way analysis of variance (ANOVA), one-way ANOVA, unpaired two-tailed Student *t* test, paired two-tailed Student *t* test). A *P* value less than 0.05 was considered statistically significant.

## Results

### KML29 improves hindpaw secondary allodynia via CB receptors in end-stage OA

**Fig. 1:**
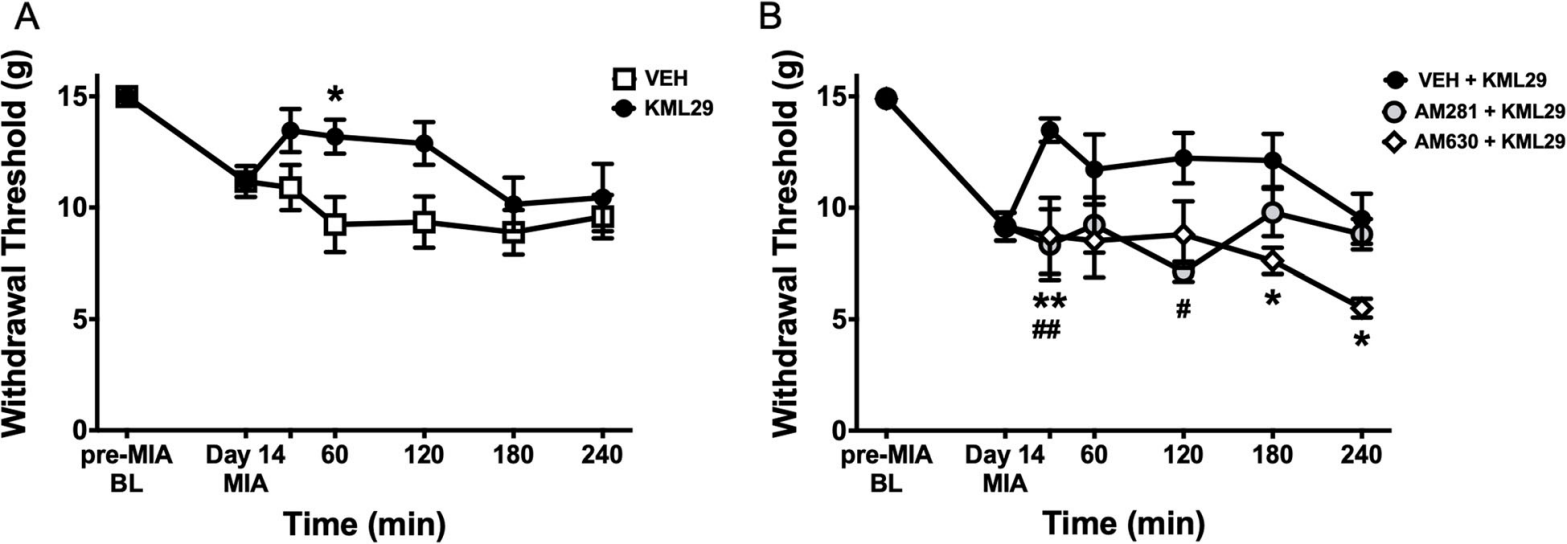
Analgesic effect of locally administered KML29 on MIA-induced secondary allodynia. On day 14 following intra-articular injection of MIA, the hindpaw withdrawal threshold was significantly reduced by local administration of KML29 (700 μg) over a 240-min time course (**a**) (*P* < 0.001; two-way ANOVA with Sidak post hoc test; *P < 0.05; *n* = 8–10). The analgesic effect of KML29 was blocked following administration of either the CB1R antagonist, AM281 (75 μg), or the CB2R antagonist, AM630 (75 μg) (**b**) (*P* < 0.0001; two-way ANOVA with Tukey post hoc test; **P < 0.01; ^##^*P* < 0.01; *P < 0.05; *n* = 6–10). Data are mean values ± SEM. ANOVA, analysis of variance; MIA, sodium monoiodoacetate; VEH, vehicle; *post hoc comparison between KML29 and VEH (**a**) and AM630 + KML29 to VEH + KML29 (**b**); ^#^post hoc comparison between AM281 + KML29 and VEH + KML29 (**b**)

In MIA-injected rats, hindpaw withdrawal threshold decreased from 14.98 ± 0.08 g on day 0 to 11.17 ± 0.70 g on day 14 (Fig. 1a). Intra-articular administration of KML29 significantly improved the hindpaw withdrawal threshold over the 240-min time period (*P* < 0.001; two-way ANOVA; *n* = 8–10).

On day 14 of the MIA model, the separate groups of animals were treated with either a CBR1 antagonist (AM281) or a CBR2 antagonist (AM630). Both antagonists significantly blocked the anti-allodynic effect of KML29 on hindpaw withdrawal threshold over the 240-min time course (*P* < 0.0001; two-way ANOVA; *n* = 6; Fig. 1b).

### Celecoxib dose-dependently improves OA pain and inflammation on day 1 of the MIA model

**Fig. 2:**
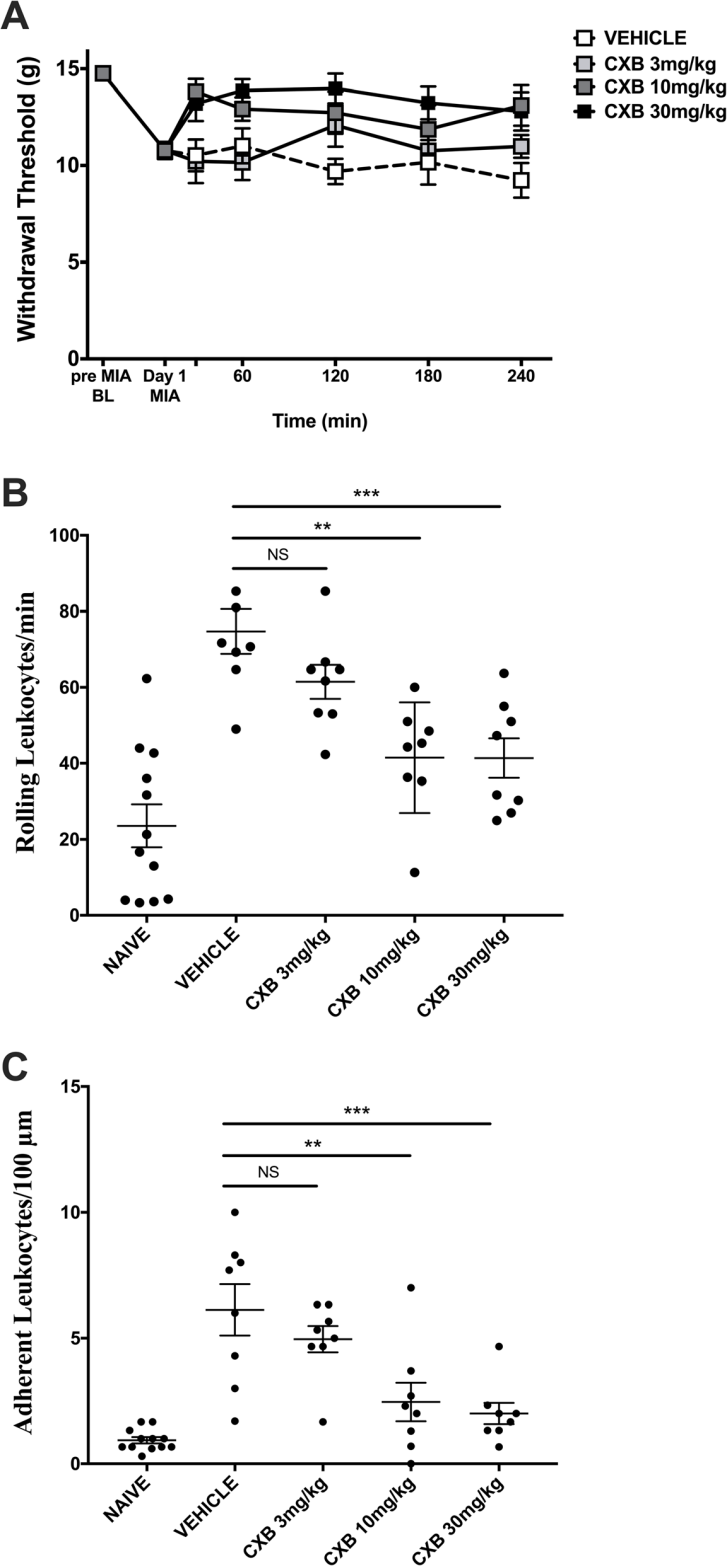
Celecoxib dose-dependently improves pain and inflammation on day 1 of MIA-induced model of OA. MIA-induced secondary allodynia was present on day 1 of the model. Systemic administration of CXB dose-dependently improved the hindpaw withdrawal threshold over a 240-min time course (**a**) (*P* < 0.0001; two-way ANOVA with Tukey post hoc test; ^##^*P* < 0.01, ^#^*P* < 0.05, *P < 0.05, ^$$^*P* < 0.01, ^$^*P* < 0.05, ^∇∇∇^*P* < 0.001, ^∇∇^*P* < 0.01, ^∇^*P* < 0.05; *n* = 8). CXB dose-dependently decreased both rolling leukocytes (**b**) (*P* < 0.0001; one-way ANOVA with Tukey post hoc test; **P < 0.01; *n* = 8) and adherent leukocytes (**c**) (*P* < 0.0001; one-way ANOVA with Tukey post hoc test; ****P < 0.0001, **P < 0.01; *n* = 8) at 360 min post-drug administration. Data are mean values ± SEM. ANOVA, analysis of variance; BL, baseline; CXB, celecoxib; MIA, sodium monoiodoacetate; VEH, vehicle; ^#^indicates post hoc comparison between 10 mg/kg and vehicle; *post hoc comparison between 3 and 10 mg/kg; ^∇^post hoc comparison between 30 mg/kg and vehicle; ^$^post hoc comparison between 3 and 30 mg/kg

On day 1 post-MIA induction, systemic administration of CXB dose-dependently improved the hindpaw withdrawal threshold (*P* < 0.0001; two-way ANOVA; *n* = 8; Fig. 2a) over the 240-min time course post-drug administration. At 360 min post-CXB injection, there was also a dose-dependent decrease in both rolling (Fig. 2b) and adherent (Fig. 2c) leukocytes (*P* < 0.0001; one-way ANOVA; *n* = 8).

### Combining KML29 and CXB reduces pain and leukocyte trafficking on day 1 MIA

**Fig. 3:**
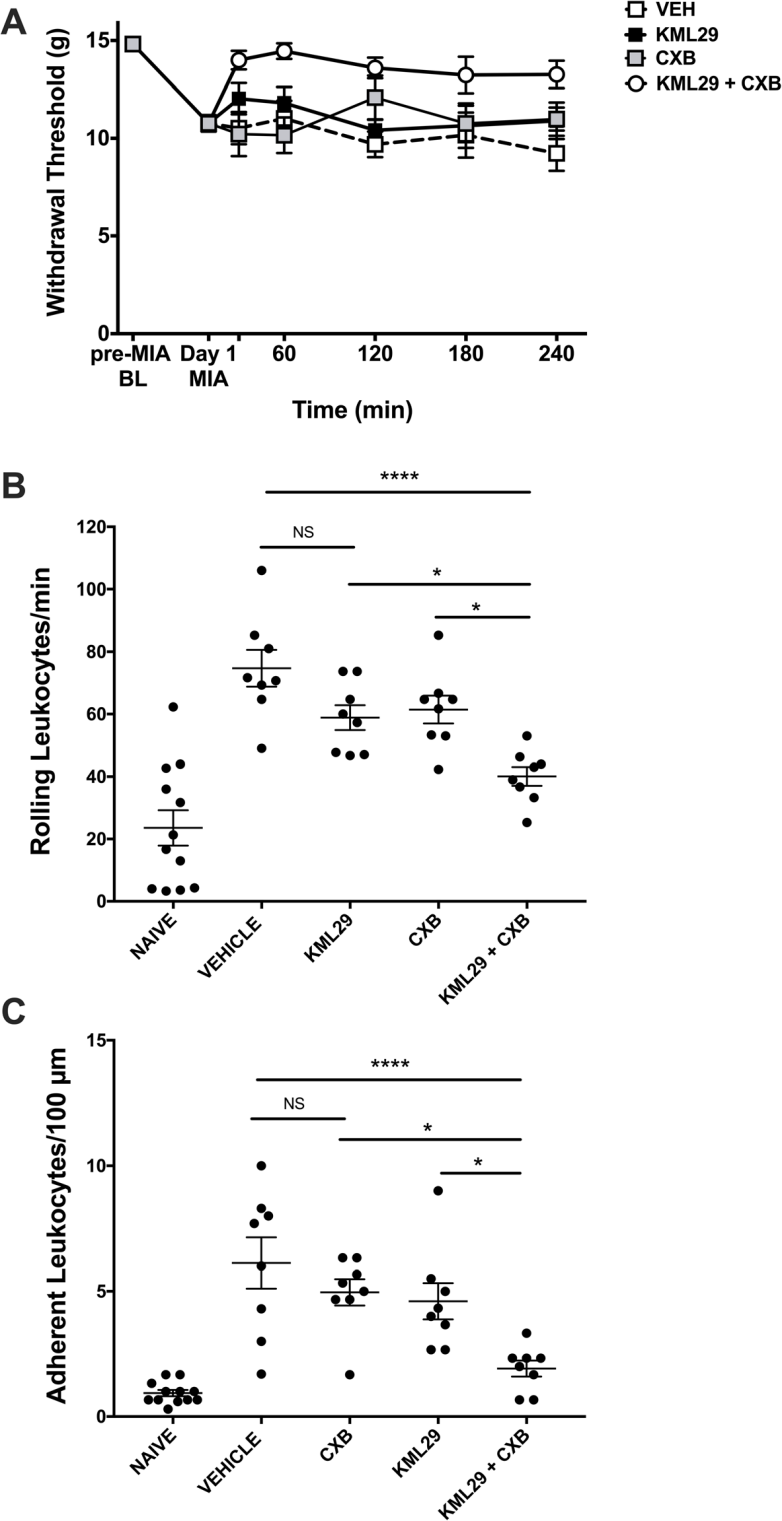
Combination of KML29 and low-dose celecoxib improves pain and inflammation on day 1 of MIA model of OA. Local administration of KML29 (700 μg) and systemic administration of CXB (3 mg/kg) significantly improved the hindpaw withdrawal threshold over a 240-min time course (**a**) compared to either treatment alone (*P* < 0.0001; two-way ANOVA with Tukey post hoc test; ***P < 0.001, **P < 0.01, *P < 0.05, ^##^*P* < 0.01, ^#^*P* < 0.05, ^∇∇∇^*P* < 0.001, ^∇∇^*P* < 0.01; *n* = 8). The KML29 and CXB combination also significantly decreased both rolling leukocytes (**b**) (*P* < 0.0001; one-way ANOVA with Tukey post hoc test; ****P < 0.0001, *P < 0.05; *n* = 8) and adherent leukocytes (**c**) (*P* < 0.0001; one-way ANOVA with Tukey post hoc test; ***P < 0.001, *P < 0.05; *n* = 8) at 360 min post-drug administration, compared to either treatment alone. Data are mean values ± SEM. ANOVA, analysis of variance; BL, baseline; CXB, celecoxib; MIA, sodium monoiodoacetate; VEH, vehicle; *post hoc comparison between KML29 + CXB and vehicle; ^#^post hoc comparison between KML29 and KML29 + CXB; ^∇^post hoc comparison between CXB and KML29 + CXB

One day post-MIA induction, KML29 reduced secondary allodynia compared to vehicle-treated animals (*P* < 0.05; two-way ANOVA; *n* = 8; Fig. 3a). The combination of KML29 and CXB had a greater anti-allodynic effect as paw withdrawal threshold was higher than all other treatment groups over the 240-min time course (*P* < 0.0001). A low dose of CXB which had no effect itself in the acute studies was chosen for the combination experiments with KML29.

With respect to joint inflammation, the combination of KML29 and CXB treatment significantly decreased rolling leukocytes from 74.71 ± 5.90 cells in vehicle-treated animals to 40.08 ± 3.01 in drug-treated rats (*P* < 0.001; one-way ANOVA; *n* = 6–8; Fig. 3b). Adherent leukocyte number also reduced from 6.13 ± 1.02 in the vehicle-treated group to 1.92 ± 0.32 leukocytes in the combination therapy group (*P* < 0.001; one-way ANOVA; *n* = 6–8; Fig. 3c). These anti-inflammatory effects were significantly greater than either CXB or KML29 alone when compared to vehicle post hoc (*P*< 0.05).

### Early treatment with KML29 and CXB prevents the development of MIA-induced hindpaw secondary allodynia

**Fig. 4:**
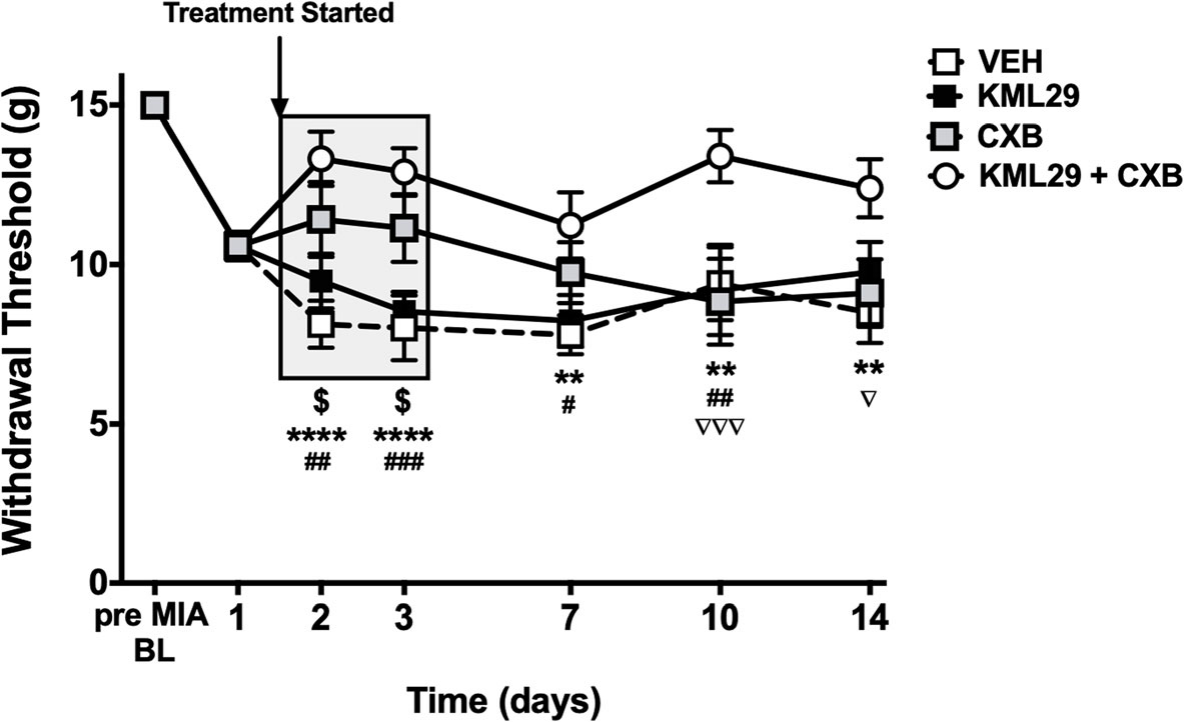
Early treatment with KML29 and celecoxib combination prevents MIA-induced secondary allodynia. Early treatment with combination of KML29 and CXB, given on days 1–3 post-MIA induction, significantly improve the hindpaw withdrawal threshold over the 14-day development of the model (*P* < 0.0001; two-way ANOVA with Tukey post hoc test; ^$^*P* < 0.05, ****P < 0.0001, **P < 0.01, *P < 0.05, ^###^*P* < 0.001, ^##^*P* < 0.01, ^#^*P* < 0.05, ^∇∇∇^*P* < 0.001, ^∇^*P* < 0.05; *n* = 8–10), compared to either treatment alone or vehicle. Data are mean values ± SEM. ANOVA, analysis of variance; BL, baseline; CXB, celecoxib; MIA, sodium monoiodoacetate; VEH, vehicle; *post hoc comparison between KML29 + CXB and vehicle; ^#^post hoc comparison between KML29 and KML29 + CXB; ^$^post hoc comparison between CXB and vehicle; ^∇^post hoc comparison between CXB and KML29 + CXB

Compared to vehicle, treating MIA rats with a combination of KML29 and CXB on days 1–3 significantly blocked MIA-induced hindpaw secondary allodynia at the end stage of OA development (*P* < 0.0001; two-way ANOVA; *n* = 8; Fig. 4). The analgesic effect of the combination therapy remained significantly greater than either CXB or KML29 alone until day 14 of the MIA model.

## Discussion

The endocannabinoid system is showing increasing promise as an effective target for the control of joint inflammation and pain [13]. Enhancing anandamide accumulation in joint tissues by inhibiting FAAH bioactivity has already been shown to ameliorate OA pain in rodent models [14, 15]. The main limitation of some of these FAAH inhibitors is that they can have off-target effects, and high levels of AEA can activate pro-algesic targets such as TRPV1 [15, 16]. Other endocannabinoids, such as 2-AG, could also have beneficial effects for the management of OA pain and inflammation although the evidence so far is limited. This study showed for the first time that local administration of the MAGL inhibitor KML29 into rat knee joints improved the hindpaw withdrawal threshold in a model of established OA. This observation corroborates a previous finding indicating that systemic administration of the MAGL inhibitor, MJN110, reduced referred pain in the MIA model [6]. Taken together, these findings suggest that MAGL blockade could act peripherally as well as centrally to reduce OA pain. The anti-allodynic effect of KML29 demonstrated here was blocked by both CB1R and CB2R antagonists confirming a cannabinoid-dependent mechanism. The involvement of cannabinoid receptors in mediating 2-AG analgesia has been described elsewhere for other MAGL inhibitors [6, 17].

Biochemical studies have found that 2-AG can be metabolised by COX-2 leading to the formation of pro-inflammatory prostaglandins [9]. This additional pathway would mitigate the full potential of 2-AG to reduce pain and inflammation. Thus, a possible stratagem to favour the anti-inflammatory and analgesic properties of 2-AG in vivo would be to combine a MAGL inhibitor with a coxib. A dose-response curve was generated for CXB to identify a “sub-clinical” dose of CXB that could be combined with KML29 to potentiate the therapeutic effects of 2-AG. Acute administration of the KML29 + CXB combination therapy on day 1 of the MIA model significantly improved pain and inflammation compared to either treatment alone. A study by Crowe et al. showed that combining the MAGL inhibitor JZL184 with the non-selective COX inhibitor diclofenac synergistically reduced both cold and mechanical allodynia in a mouse model of neuropathic pain. These findings support the approach that combining a MAGL inhibitor with a COX inhibitor can provide greater symptom relief than the individual drugs alone.

Having established an anti-inflammatory and analgesic effect of MAGL and COX-2 inhibition acutely, experiments were undertaken to test the effect of early treatment with KML29 and CXB on OA progression in the MIA model. Previously, it has been demonstrated that prophylactic administration of the phytocannabinoid CBD [11] or the FAAH inhibitor URB597 [14] have the ability to reduce pain development at later stages of the MIA model of OA. Prophylactic treatment of MIA-injected animals in the early inflammatory phase of the model (days 1–3) with the combination of KML29 and CXB prevented MIA-induced mechanical allodynia at day 14 compared to vehicle or either treatment alone. Hence, blocking 2-AG hydrolysis in conjunction with COX-2 inhibition produced an effective anti-allodynic effect in the later stages of the MIA model. The doses of drugs were chosen because they had little to no effect on joint pain by themselves suggesting that a combined effect of the two therapies was occurring in the OA knee. One clinical advantage of this combined effect is that it would allow for a reduction in coxib dosing in patients with chronic arthritis. Long-term coxib use has been linked with numerous negative side effects including gastrointestinal bleeding, cardiovascular toxicity, and liver and kidney dysfunction. By combining a low-dose coxib with a low dose of a MAGL inhibitor, it may be possible to improve the safety profile of the coxib by limiting the dose necessary to elicit pain relief in OA. Other investigations have shown that MAGL inhibition can impart gastroprotection in the diclofenac-induced model of gastric haemorrhage in mice [8, 18]. This observation as well as the findings presented here suggests that the addition of a MAGL inhibitor to a classic coxib treatment regimen has the potential to mitigate the adverse side effects observed with NSAIDs alone.

The acute inflammation and neuropathy associated with the MIA model likely contribute to joint pathology and degeneration. The steroid fluocinolone and the NSAID meloxicam have been found to reduce joint damage in the MIA model [19] suggesting that early treatment with anti-inflammatories could stave off disease progression. Various nutraceuticals such as avocado soybean unsaponifiables and pomegranate juice have also been found to be chondroprotective in MIA animals [20, 21]. The anti-inflammatory effect of KML29 described here suggests that endocannabinoids could avert OA degeneration; however, this requires further investigation.

## Conclusions

In summary, the present study identified a novel mechanism by which acute MAGL inhibition can reduce pain via a cannabinoid receptor mechanism. Since KML29 was administered intra-articularly, these findings demonstrate that the MAGL inhibitor was acting peripherally which complements other reports of a systemic mode of action to reduce pain. Furthermore, KML29 and CXB were shown to work together in the joint to alleviate OA pain and inflammation. This combination therapy may be an effective treatment strategy to mitigate osteoarthritis-associated pain and allow a reduction in coxib dosing.

## Data Availability

The datasets used and/or analysed during the current study are available from the corresponding author on reasonable request.

## Abbreviations

2-AG: 2-Arachidonoylglycerol
AEA: Anandamide
ANOVA: Analysis of variance
CBR1: Cannabinoid 1 receptor
CBR2: Cannabinoid 2 receptor
COX-2: Cyclooxygenase-2
CXB: Celecoxib
ECS: Endocannabinoid system
FAAH: Fatty acid amide hydrolase
i.artic.: Intra-articular
i.p.: Intraperitoneal
IVM: Intravital microscopy
MAGL: Monoacylglycerol lipase
MIA: Sodium monoiodoacetate
NSAID: Non-steroidal anti-inflammatory drug
OA: Osteoarthritis
s.c.: Subcutaneous
SEM: Standard error of the mean
VEH: Vehicle

## References

[1] Fu K, Robbins SR, McDougall JJ (2018). Osteoarthritis: the genesis of pain. Rheumatology (Oxford).

[2] Sokolove J, Lepus CM (2013). Role of inflammation in the pathogenesis of osteoarthritis: latest findings and interpretations. Ther Adv Musculoskelet Dis.

[3] Dray A (2008). New horizons in pharmacologic treatment for rheumatic disease pain. Rheum Dis Clin N Am.

[4] van Laar M, Pergolizzi JV, Mellinghoff HU, Merchante IM, Nalamachu S, O’Brien J (2012). Pain treatment in arthritis-related pain: beyond NSAIDs. Open Rheumatol J.

[5] Savinainen JR, Jarvinen T, Laine K, Laitinen JT (2001). Despite substantial degradation, 2-arachidonoylglycerol is a potent full efficacy agonist mediating CB(1) receptor-dependent G-protein activation in rat cerebellar membranes. Br J Pharmacol.

[6] Burston JJ, Mapp PI, Sarmad S, Barrett DA, Niphakis MJ, Cravatt BF (2016). Robust anti-nociceptive effects of monoacylglycerol lipase inhibition in a model of osteoarthritis pain. Br J Pharmacol.

[7] Guindon J, Guijarro A, Piomelli D, Hohmann AG (2011). Peripheral antinociceptive effects of inhibitors of monoacylglycerol lipase in a rat model of inflammatory pain. Br J Pharmacol.

[8] Crowe Molly S, Leishman Emma, Banks Matthew L, Gujjar Ramesh, Mahadevan Anu, Bradshaw Heather B, Kinsey Steven G (2015). Combined inhibition of monoacylglycerol lipase and cyclooxygenases synergistically reduces neuropathic pain in mice. British Journal of Pharmacology.

[9] Di Marzo V (2008). Endocannabinoids: synthesis and degradation. Rev Physiol Biochem Pharmacol.

[10] Chaplan SR, Bach FW, Pogrel JW, Chung JM, Yaksh TL (1994). Quantitative assessment of tactile allodynia in the rat paw. J Neurosci Methods.

[11] Philpott HT, O’Brien M, McDougall JJ (2017). Attenuation of early phase inflammation by cannabidiol prevents pain and nerve damage in rat osteoarthritis. Pain..

[12] Andruski B, McCafferty DM, Ignacy T, Millen B, McDougall JJ (2008). Leukocyte trafficking and pain behavioral responses to a hydrogen sulfide donor in acute monoarthritis. Am J Physiol Regul Integr Comp Physiol.

[13] O’Brien M, McDougall JJ (2018). Cannabis and joints: scientific evidence for the alleviation of osteoarthritis pain by cannabinoids. Curr Opin Pharmacol.

[14] McDougall JJ, Muley MM, Philpott HT, Reid A, Krustev E (2017). Early blockade of joint inflammation with a fatty acid amide hydrolase inhibitor decreases end-stage osteoarthritis pain and peripheral neuropathy in mice. Arthritis Res Ther.

[15] Malek N, Mrugala M, Makuch W, Kolosowska N, Przewlocka B, Binkowski M (2015). A multi-target approach for pain treatment: dual inhibition of fatty acid amide hydrolase and TRPV1 in a rat model of osteoarthritis. Pain..

[16] Kawahara H, Drew GM, Christie MJ, Vaughan CW (2011). Inhibition of fatty acid amide hydrolase unmasks CB1 receptor and TRPV1 channel-mediated modulation of glutamatergic synaptic transmission in midbrain periaqueductal grey. Br J Pharmacol.

[17] Ghosh S, Wise LE, Chen Y, Gujjar R, Mahadevan A, Cravatt BF (2013). The monoacylglycerol lipase inhibitor JZL184 suppresses inflammatory pain in the mouse carrageenan model. Life Sci.

[18] Kinsey SG, Wise LE, Ramesh D, Abdullah R, Selley DE, Cravatt BF (2013). Repeated low-dose administration of the monoacylglycerol lipase inhibitor JZL184 retains cannabinoid receptor type 1-mediated antinociceptive and gastroprotective effects. J Pharmacol Exp Ther.

[19] TenBroek EM, Yunker L, Nies MF, Bendele AM (2016). Randomized controlled studies on the efficacy of antiarthritic agents in inhibiting cartilage degeneration and pain associated with progression of osteoarthritis in the rat. Arthritis Res Ther..

[20] Al-Afify ASA, El-Akabawy G, El-Sherif NM, El-Safty FEA, El-Habiby MM (2018). Avocado soybean unsaponifiables ameliorates cartilage and subchondral bone degeneration in mono-iodoacetate-induced knee osteoarthritis in rats. Tissue Cell.

[21] Hadipour-Jahromy M, Mozaffari-Kermani R (2010). Chondroprotective effects of pomegranate juice on monoiodoacetate-induced osteoarthritis of the knee joint of mice. Phytother Res.

